# Fractionation for further conversion: from raw corn stover to lactic acid

**DOI:** 10.1038/srep38623

**Published:** 2016-12-05

**Authors:** Ting He, Zhicheng Jiang, Ping Wu, Jian Yi, Jianmei Li, Changwei Hu

**Affiliations:** 1 Key Laboratory of Green Chemistry and Technology, Ministry of Education, College of Chemistry, Sichuan university, Chengdu, Sichuan 610064, China

## Abstract

Fractionation is considered to be one promising strategy to utilize raw biomass to its fullest and produce chemicals with high selectivity. Herein, ethanol/H_2_O (1/1, v/v) co-solvent with 0.050 M oxalic acid is used to simultaneously fractionate 88.0 wt% of hemicellulose and 89.2 wt% of lignin in corn stover, while cellulose is not obviously degraded. H_2_O dissolves hemicellulose, G unit and those with β-O-4 linkage of lignin; whereas ethanol extracts G and S units as well as the skeleton with β-5 and β-β linkages of lignin. Oxalic acid effectively catalyzes the hydrolysis of hemicellulose and breaks the intermolecular linkages between hemicellulose and lignin, therefore further promotes the release of lignin. The dissolved hemicelluloses derivatives are reprocessed to produce lactic acid obtaining a high yield of 79.6 wt% with 90% selectivity by the catalysis of MgO. The remained cellulose and recovered lignin can be used further as feedstock to produce chemicals.

Recently, increasing interest has been focusing on the production of chemicals and fuels from renewable lignocellulosic biomass, due to the depletion of fossil resource and accompanying environmental pollution^1^,^2^,^3^. Hemicellulose, lignin and cellulose are the three main components of lignocelluloses. Utilization of the three main components without discard is of significant importance to fully exploit the potential of lignocellulose. Since these components exhibit distinct structure, numerous kinds of chemicals can be obtained from the simultaneous conversion of the three components in lignocelluloses^4^,^5^, leading to difficulties in their utilization, separation and further refinery. That is to say, the selectivity to each target product is quite low.

Fractionation provides ways to produce chemicals with high selectivity and to use the raw lignocellulose to its fullest^6^,^7^,^8^,^9^,^10^. Many fractionation methods have been developed for biorefinery concept, in most cases, aiming at providing carbohydrate fractions (cellulose and hemicellulose) for the following fermentation processes. In fact, lignin and hemicellulose are usually simultaneously extracted, leaving cellulose component apart, because the solvolysis of highly structured cellulose usually needs harsher conditions^4^,^11^,^12^. The lignocellulosic biomass can be then fractionated into its three components, which generate three feedstock streams for futher conversion. For the possibility to avoid harsh conditions (e.g. strong acids or alkaline) and complex processes, organic acid-catalyzed organosolv methods are promising to fractionate the three components in a more economical and environmentally friendly way^13^,^14^. Typically, a so-called “organocat process” was reported by Leitner *et al*., in which oxalic acid selectively catalyzed the depolymerization of hemicellulose and the dissolution of lignin in a 2-methyltetrahydrofuran-water biphasic system^15^. This process released >80% of hemicellulose as sugars from beech wood with 0.1 M oxalic acid at 140 °C. The amount of recovered lignin reached up to ~60–70% of the theoretical value from the average beech wood composition, whereas most of the cellulose was remained in solid pulp. However, the performance of both solvent and catalyst on the dissolution of the components is still unclear, which needs to be further investigated. Moreover, unlike cellulose and lignin, the fractionated hemicellulose derivatives by acid-catalyzed solovlysis is usually difficult to be separated to obtain solid intermediates, due to its further degradation into small molecular products. Thus, it is relatively hard to store and transport for further use as feedstock. Therefore, the prompt conversion of dissolved hemicellulose derivatives to chemicals with high selectivity after fractionation can be a way to avoid the problem.

Lactic acid, regarded as one of the top biomass derived platform chemicals by the US Department of Energy (DOE), has been widely used in food, pharmaceutical and chemical industries, especially in biodegradable polymer polylactic acid (PLA) production^16^,^17^,^18^. More than 90% of lactic acid are produced via fermentation of carbohydrates, however, the biotechnological routes are high costly with low productivity^17^,^19^,^20^. In addition, the bioconversion process cannot be directly applied to the more available actual biomass without pretreatment, because of the inhibition of lignin^21^,^22^. At present, many efforts have been transferring to explore new chemo-catalytic methods in the interest of establishing a less expensive route for producing lactic acid^23^,^24^,^25^. Fangming Jin and coworkers obtained lactic acid (<42% carbon yield) from cellulose in the presence of alkaline^26^,^27^. Yanliang Wang and coworkers obtained a yield of lactic acid (68 wt%) from cellulose in the presence of 7 mM Pb(II)^28^. ErCl_3_ showed an excellent catalytic activity to produce lactic acid from pure cellulose with a high yield of 91.9% (carbon yield)^29^. Although satisfied yield of lactic acid is obtained, these homogeneous catalysts somehow encounter the environment pollution and recycle problems, which hinders their application in industry. Heterogeneous catalyst therefore becomes an appropriate alternative. Glucose and cellulose (with C_6_ sugars units) are commonly used as the resources to produce lactic acid. To date, the hemicelluloses (mainly with C_5_ sugar units), especially those from actual lignocellulose, are seldom employed directly to produce lactic acid. Hongfei Lin *et al*. Reported that xylose and xylan could be used as resources to produce lactic acid by the catalysis of ZrO_2_. However, the highest yield of lactic acid was only 42 mol% from xylose^30^. Actually, the hemicellulose via pretreatment process is a potential material to produce chemicals^31^,^32^,^33^,^34^,^35^. In this work, we therefore developed an integrated route to efficiently separate the three components of corn stover under mild conditions and prepare lactic acid from the derived-hemicellulose with high yield and selectivity.

## Results and Discussion

**Figure 1:**
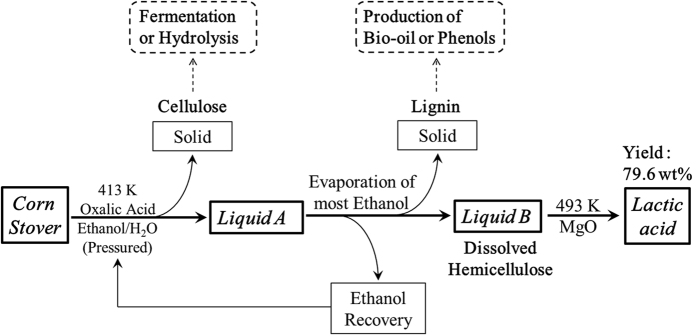
The schematic process for the fractionation of corn stover and the production of lactic acid.

A novel integrated route was developed to successfully fractionate corn stover into its three main components, and to convert the hemicellulose derivatives selectively to lactic acid (as shown in Fig. 1). This approach firstly disentangled both hemicellulose and lignin from corn stover in ethanol/H_2_O co-solvent with oxalic acid as catalyst. After cellulose pulp and lignin precipitate were orderly separated, the dissolved hemicellulose derivatives in aqueous solution was selectively converted to lactic acid by the catalysis of MgO.

### Simultaneous dissolution of hemicellulose and lignin

Our initial experiments aimed to explore the effect of solvent (ethanol and H_2_O) on the conversion of corn stover in the absence of catalyst (Entry 1–5 in Table 1). We observed that the conversion of hemicellulose in hot pressurized water (initial pressure of N_2_ was 2 MPa before heating) reached up to 44.5 wt% at 140 °C (Entry 1 in Table 1). Hot pressurized water could selectively extract hemicellulose (autohydrolysis process), not only playing an acid catalytic role to cut the links between hemicellulose and lignin as well as between carbohydrates, but also dissolving the hemicellulose fragments. A little amount of xylose and acetic acid were detected by HPLC. It indicated that water also partly broke the intramolecular linkages in hemicellulose fragments, resulting in the generation of small molecular compounds. Less water in ethanol/H_2_O co-solvent led to lower conversion of hemicellulose. In agreement with our previous report^36^, the presence of ethanol in the system was beneficial to lignin dissolution. When the concentration of ethanol (CE, volume concentration of ethanol in H_2_O) increased from 0 to 0.5, the conversion of lignin increased from 27.5 to 38.6 wt%. However, the decrease of lignin release was observed when the CE was more than 0.5. Only 16.9 wt% of lignin was dissolved in ethanol, which was obviously lower than that obtained in water and ethanol/H_2_O (1/1, v/v) co-solvent systems. It indicated that when a part of hemicellulose (15.8~44.5 wt%) depolymerized with CE < 0.7, the linkages between hemicellulose and lignin were broken, thereby lignin was easily released from biomass matrix.

**Figure 2:**
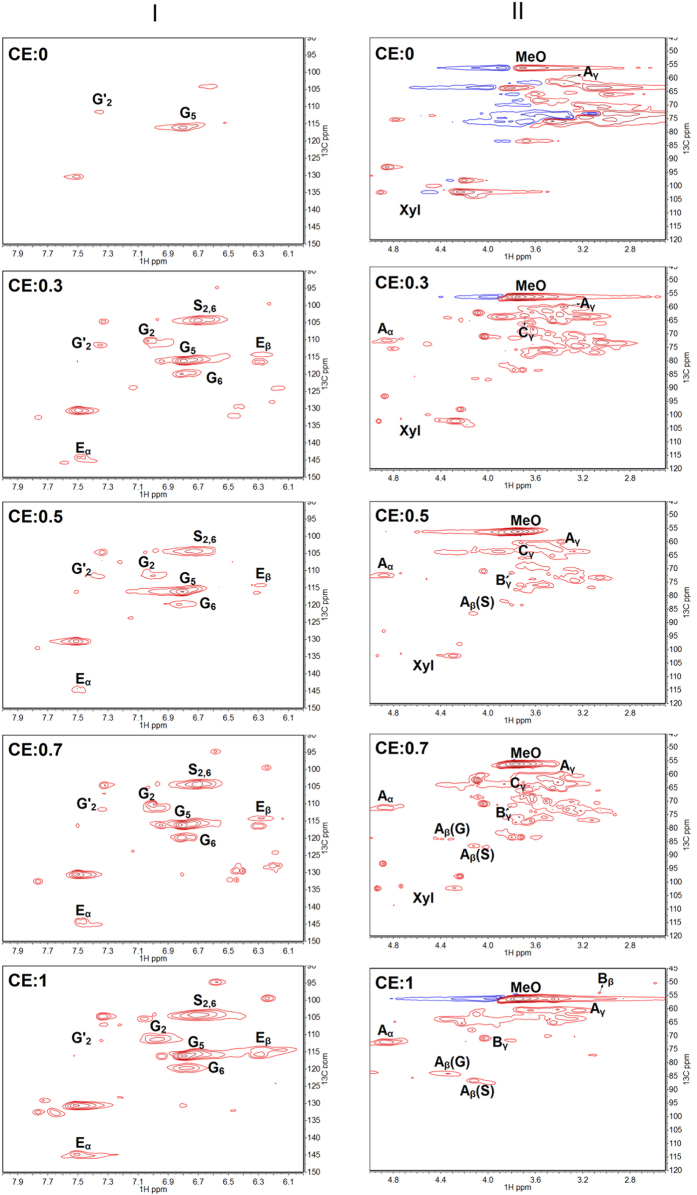
The 2D HSQC NMR analysis of liquid fraction obtained from different reaction system with various concentration of ethanol in H_2_O. (I) Aromatic C-H correlation region; (II) Aliphatic C-H correlation region. CE = the concentration of ethanol in ethanol/H_2_O solvent; A: β-O-4 linkage; B: β-β linkage; C: β-5 linkage; E: cinnamate structure; G: guaiacyl units; S: syringly units; MeO: methoyls; Xyl: xylan derived from hemicellulose.

**Figure 3:**
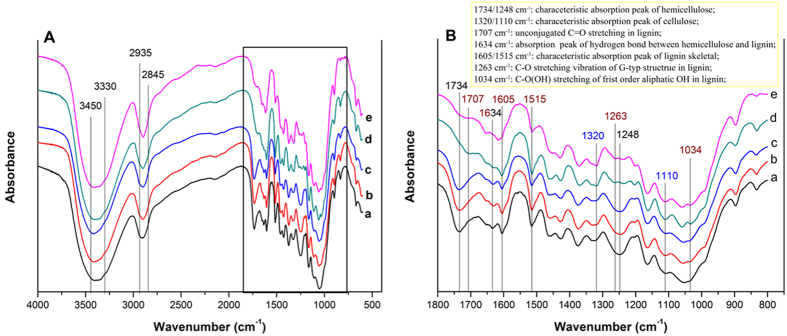
FTIR spectra of solid samples. (**A**) Full spectrum from 4000–600 cm^−1^; (**B**) Partial spectrum from 1800–800 cm^−1^. (a) Corn stover; (b) H_2_O, 0 M oxalic acid; (c) ethanol/H_2_O = 1/1, 0 M oxalic acid; (d) H_2_O, 0.050 M oxalic acid; (e) ethanol/H_2_O = 1/1, 0.050 M oxalic acid.

Without catalyst, the small molecular products derived from hemicellulose and lignin were hardly observed after treatments, suggesting that the released hemicellulose and lignin were almost oligomers. Figure 2 shows the 2D HSQC of liquid fractions obtained from different solvent systems with varied CE in the absence of catalyst (reactions of Entry 1–5 in Table 1). Supplementary Table S1 lists the attribution of the main cross-signals according to literature^36^,^37^, and the hypothetical structure of lignin-derived species is displayed in Fig. S1. The signals of lignin oligomers and derivatives from hemicellulose were observed. In H_2_O (CE = 0), the signals of xylan were observed obviously, and only G unit (guaiacyl unit) with A linkage (β-O-4 linkage) of lignin could be easily dissolved. This further proved that H_2_O was favorable for hemicellulose dissolution. In the presence of ethanol, the signals of S (syringyl unit) and E (cinnamate structure) were found. Both G and S units increased with increasing CE. Moreover, in the aliphatic C-H correlation region, the signals of A linkage gradually increased; and the signals of C (β-5 linkage) and B (β-β linkage) appeared and gradually strengthened, indicating that the units with β-5 and β-β linkages were dissolved with the addition of ethanol. Therefore, we considered that the improved dissolution of lignin could be partly ascribed to the better dissolubility of lignin G and S units as well as those with A, B and C linkages in ethanol/H_2_O co-solvent than in H_2_O. However, the signal of p-hydroxyphenyl unit (H) was not observed, implying that it was hard to dissolve H unit in the absence of catalyst. In addition, according to the FTIR spectra of solid samples (***c*** and ***e*** in Fig. 3B), we found that the intensity of peaks at 1605 and 1515 cm^−1^ decreased, which indicated that ethanol in the system greatly affected the dissolution of lignin skeletal. If only water was used as solvent, it was hard to realize the collapse of lignin skeletal even in the presence of oxalic acid (***b*** and ***d*** in Fig. 3B). So, it seems that the skeletal is one of the stubborn and difficultly dissolved parts in lignin, and ethanol provides good ability for the extraction of lignin skeletal chunks. It explained why the available dissolution of lignin successfully proceeded only in the presence of ethanol. In general, water in the extraction medium firstly broke the links among the three components, then dissolved hemicellulose derivatives and G-type lignin unit, and guaranteed the transport of ethanol into the lignocellulosic matrix, thereby enabling lignin extraction. Ethanol effectively extracted the lignin (mainly focused on lignin skeleton) and was helpful for dissolving lignin-derivatives.

Oxalic acid, as a dicarboxylic acid, has attracted increasing interests because of its good catalytic ability for polysaccharide depolymerization^15^,^38^,^39^. With the addition of 0.017 M oxalic acid, the conversion of hemicellulose and lignin greatly increased from 15.8 to 84.0 wt% and 38.6 to 83.0 wt%, respectively. In order to investigate the performance of oxalic acid on the hemicellulose and lignin conversion more detailedly, lower concentrations of oxalic acid (0.005 and 0.010 M) were also tested. We found that when it was less than 0.017 M, the concentration of oxalic acid had an obvious effect on the conversion of both hemicellulose and lignin. With further increase of oxalic amount, the conversion of both hemicellulose and lignin only slightly improved. Therefore, the addition of oxalic acid greatly improved the extraction efficiency of both hemicellulose and lignin in corn stover, especially in ethanol/H_2_O co-solvent. However, the cross-signals of H unit (in lignin) appared only when the concentration of oxalic acid was more than 0.033 M, and increased with the concentration of oxalic acid (Supplementary Fig. S2). Due to its poor dissolubility, H unit seems to be dissolved only in the presence of relatively a large amount of oxalic acid. Although the extraction efficiency of both hemicellulose and lignin was satisfied, higher concenteation (>0.033 M) of oxalic acid was tested, for the purpose of releasing the H unit. Approximately 90 wt% of both hemicellulose and lignin could be finally extracted in ethanol/H_2_O (1/1, v/v) co-solvent with 0.050 M oxalic acid at 140 °C for 1 h (Entry 11–13 in Table 1), while no significant degradation of cellulose (<8 wt%) was attained. To simultaneously convert hemicellulose and lignin while keep cellulose as much as possible, the reaction temperature and reaction time were optimized (Supplementary Fig. S3). It was obvious that the conversion of the three components increased with rising temperature. When the temperature was raised from 100 to 140 °C, the conversion of both hemicellulose and lignin increased rapidly from approximately 40 to 90 wt%, while the conversion of cellulose slightly increased from 5 to 8 wt%. Higher temperature than 140 °C obviously boosted the conversion of cellulose from 8 wt% at 140 °C to 15 wt% at 180 °C (as shown in Supplementary Fig. S3A). The bands at 1427, 1376 and 897 cm^−1^ in FTIR spectra of solid residue after treatment at 160 °C, corresponding to the crystalline structure of cellulose, obviously decreased, implying the depolymerization of cellulose in crystalline regions (Supplementary Fig. S4)^40^. These results agreed well with the literature reporting that dicarboxylic acids could selectively remove amorphous hemicellulose without extensive cellulose degradation when the pretreated temperature ranged in 80–140 °C, while the cellulose in crystalline regions would be depolymerized at temperature higher than 160 °C^15^,^38^,^41^. Similarly, longer reaction time obviously promoted the conversion of cellulose (as shown in Supplementary Fig. S3B). Therefore, we chose 0.050 M oxalic acid, 140 °C and 1 h as the optimal reaction conditions.

The raw material and solid residues were characterized by FTIR spectroscopy. The stretching vibrations of OH, CH_3_ and CH_2_ groups in the three main components of corn stover occur at 3450–3300, 2935 and 2845 cm^−1^, respectively (Fig. 3A). The main difference of FTIR spectra was in the regions ranged from 1800–800 cm^−1^ (Fig. 3B). In the presence of oxalic acid (***d*** and ***e***), the peaks at 1320 and 1110 cm^−1^ (assigned to cellulose) increased, while the peaks at 1734 and 1248 cm^−1^ (assigned to hemicellulose) and those at 1707 and 1263 cm^−1^ (corresponding to lignin) obviously weakened. This showed that the significant improvement of cellulose content in residues as well as the dissolution of both hemicellulose and lignin from corn stover was attributed to the presence of oxalic acid. The weakened peaks at 1034 cm^−1^ (attributing to C-O(H) stretching vibration of the first order aliphatic OH in lignin)^42^ and 1634 cm^−1^ (attributing to a hydrogen bond between hemicellulose and lignin)^43^ suggested the damage of hydrogen bond between hemicellulose and lignin, resulting from the penetration of oxalic acid in the amorphous region of the biomass^44^. With 0.050 M oxalic acid, 47.0 wt% of lignin was degraded in water (Entry 7 in Table 1). Most of the G-type lignin (at 1263 cm^−1^) was removed according to ***d*** of Fig. 3B. In addition, we could find obvious signals of G and S units as well as those with A linkage from its 2D HSQC (Supplementary Fig. S5), indicating the release of G and S lignin with A linkage. It seemed that the G-type lignin was more easily released from biomass matrix, and S-type lignin tended to be released by the catalysis of oxalic acid in H_2_O. We considered that the first order aliphatic C-OH in lignin was intimately related to the formation of the hydrogen bond between hemicellulose and lignin. The oxalic acid led to the breakage of the hydrogen bond, effectually contributing to the dissolution of G lignin. Therefore, the facilitating of the conversion of both hemicellulose and lignin by oxalic acid could be ascribed to the breakage of interaction between them. Control experiments with different catalysts (Supplementary Fig. S6) showed that other acids, including inorganic acid (hydrochloric acid) and organic acids (butanedioic acid, formic acid, acetic acid and maleic acid), with approximately the same initial pH value as oxalic acid, resulted in close conversion of both hemicellulose and lignin. Potassium oxalate with the same concentration of oxalate exhibited poor catalytic activity for the conversion of both hemicellulose and lignin. Therefore, moderate acidity (pH = 2) of oxalic acid in the system was the main factor that resulted in the simultaneous dissolution of the hemicellulose and lignin without significantly affecting the cellulose component.

### Separation of the three components

**Figure 4:**
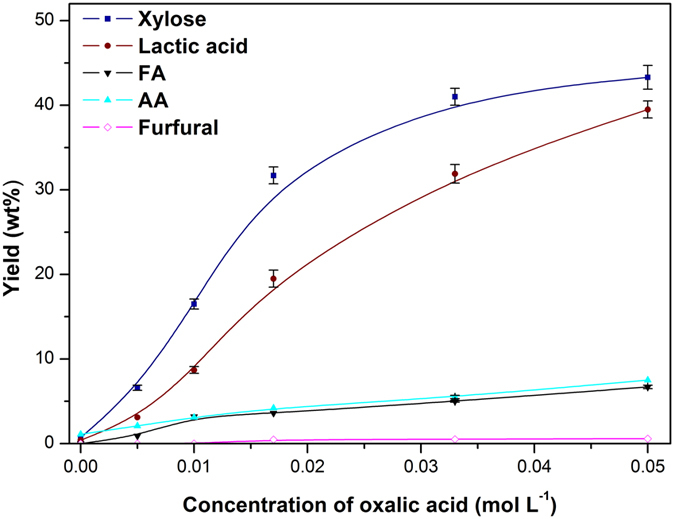
The yield of main monomers with different concentration of oxalic acid. Reaction conditions: 3.0 g corn stover, ethanol/H_2_O (1/1, v/v) 100 mL, 140 °C. 1 h, initial pressure 2 MPa N_2_.

Under the cooperative action of catalyst and solvent, the cellulose in corn stover lost the protecting shield of hemicellulose and lignin (Supplementary Fig. S7A). It would be more available for biodegradation by cellulolytic enzymes. The majority of cellulose was remained in residue with a purity of ~70 wt%. Because the removed hemicellulose and lignin were in amorphous region, the CI (crystalline) of resulted cellulose was increased from 79% to 88% (Supplementary Fig. S7B). Nevertheless, the majority (80%) of “others” in corn stover, including ash, extractives and unknown composition, was retained in residue with cellulose. After evaporation of ethanol from the liquid (Liquid A, referring to Fig. 1), a yield of 83 wt% (based on converted lignin) lignin (a rufous powder) could be recovered. Its 2D HSQC NMR spectra (Supplementary Fig. S8) showed that the main construction unit of lignin was remained. The separated lignin was dsisolved in THF and analyzed by GPC. Its M_w_ (1480 g mol^−1^) was much lower than that in natural raw material, resulting from the fractionation and dissolution treatments. However, the relevant small molecular products (*e*.*g*. monophenic compounds) derived from lignin was hardly detected. Thus, it indicated that the removed lignin was mainly degradated to oligomers (~10 benzene propane units) without any monomers. Many small molecular products were detected in hemicellulose fraction (Liquid B) (Fig. 4), including xylose, lactic acid, some (<9 wt%) formic acid (FA) and acetic acid (AA), while furfural was rarely observed. The yields of lactic acid and xylose were greatly improved with increasing concentration of oxalic acid, and 43.3 wt% of xylose and 39.5 wt% of lactic acid were obtained in the presence of 0.050 M oxalic acid. It is commonly accepted that xylan chain is firstly decomposed to xylo-oligosaccharides, and then the decomposition proceeds to yield xylose monomer^45^. Increasing the concentration of oxalic acid promoted the further degradation of xylo-oligosaccharides, and the resulted xylose could be further converted to other smaller molecular products like lactic acid.

### Enhancement of lactic acid production

Besides 39.5 wt% of lactic acid, 43.3 wt% of xylose was also contained in Liquid B (Table 2). We afterwards tried to further increase the yield of lactic acid through reprocessing the aqueous hemicellulose derivatives. Primarily, Liquid B was directly heated to higher temperature (160, 180 and 220 °C), and the results were shown in Table 2. At 160 °C, the yield of both xylose and lactic acid increased to 50.9 wt% and 44.9 wt%, respectively. It implied that the appropriate increase in temperature was advantageous for the further hydrolysis of xylo-oligosaccharides, resulting in the increase of xylose yield. This further proved the presence of xylo-oligosaccharides from hemicellulose conversion. The main by-products, including FA, AA and furfural, were also slightly increased. However, with further raising reaction temperature to 220 °C, the yield of both xylose and lactic acid decreased to 1.2 wt% and 14.5 wt%, respectively; while the yield of furfural significantly increased from 0.6 to 21.0 wt%. This was attributed to the dehydration of xylose to furfural at high temperatures under BrØnsted acidic ambiance, and the further decomposition of lactic acid to smaller molecular acids (like FA and AA)^30^.

**Figure 5:**
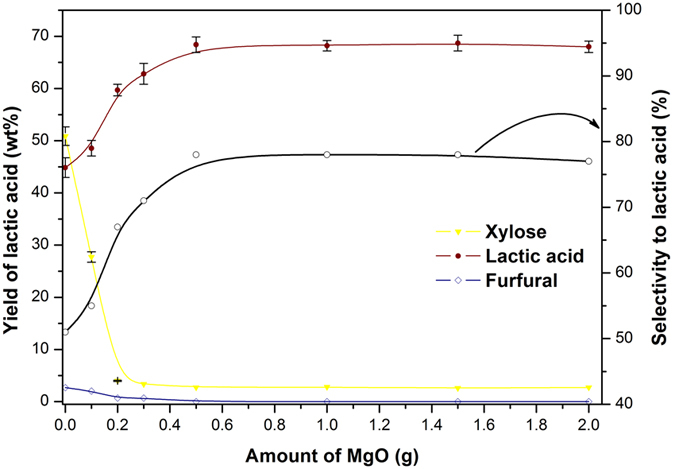
The influence of MgO amount on the yield of liquid products and selectivity to lactic acid. Reaction condition: 160 °C, 1 h, initial pressure 2 MPa N_2_.

Known that lactic acid was the main product from sugars by the catalysis of alkaline catalyst^24^, we next introduced commercially accessible MgO (with a surface area of 34 m^2^ g^−1^) to reprocess Liquid B. CO_2_ TPD results showed that a large amount of weakly basic sites and some medium/strong basic sites existed on MgO (Supplementary Fig. S9). It is believed that the weakly basic sites are the catalytic active sites, and more weakly sites there are, higher catalytic activity the MgO has^46^. SEM and TEM were also employed to characterize the structure of MgO (Supplementary Figs S10 and S11). In general, nanosheets of the MgO gather together with some nanorods insertion. In the presence of MgO, xylose was completely converted, and the yield and selectivity to lactic acid were significantly improved at 160 °C (Fig. 5). The yield of lactic acid increased to 48.6 wt% with 0.1 g MgO. Further increase of MgO amount led to obvious enhancement of lactic acid yield, which reached to 59.7 wt% when 0.2 g MgO was used. A high yield (68.4 wt%) and selectivity (78%) to lactic acid were achieved with 0.5 g MgO. However, further increase of MgO amount did not have an obvious contribution to the improvement of lactic acid yield. Note worthily, furfural was hardly detected in the presence of MgO. It was considered that MgO might change the acidic ambiance of the reaction system, so that the formation of furfural was inhibited, whereas the conversion of xylose to lactic acid was effectively facilitated.

**Figure 6:**
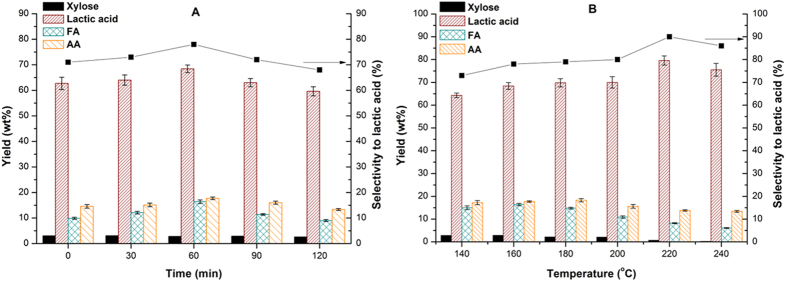
The effect of reaction time (**A**) and temperature (**B**) on the formation of lactic acid.

As the reaction time extended, the yield of lactic acid initially increased but decreased afterwards at 160 °C with 0.5 g MgO (Fig. 6A). When t = 0 (stopping heating the reactor immediately as soon as it reached to the designed temperature), the yield of lactic acid was 62.7 wt%, and the highest yield reached 68.4 wt% at t = 1 h. Futher increasing reaction time did not lead to further conversion of xylose with no obvious change in the yield of FA and AA. The production of lactic acid was further conducted at different reaction temperatures (Fig. 6B). When the reaction temperature was raised to 200 °C from 140 °C, the yield of lactic acid had a slight increase, from 64.3 to 69.8 wt%. With further increase of temperature to 220 °C, nearly all the xylose was converted, and the yield and selectivity to lactic acid got the maximum, 79.6 wt% and 90%, respectively. Thus, it was considered that MgO could inhibit the lactic acid from further conversion at higher temperature. However, a drop of lactic acid yield happened at 240 °C. Therefore, the reaction temperature should be maintained below 240 °C to restrain further conversion of lactic acid.

We also studied the recyclability of MgO. As shown in Supplementary Fig. S12A, its catalytic activity for lactic acid production had no obvious loss after 5 runs. It indicated that commercial MgO indeed exhibited excellent recyclability. A little change of MgO was observed after reuse through XRD analysis (Supplementary Fig. S12B). Because of its advantages such as safety, low cost and easy preparation, MgO is a potential industrial catalyst for the production of lactic acid.

### Discussion on the origin of lactic acid

**Figure 7:**
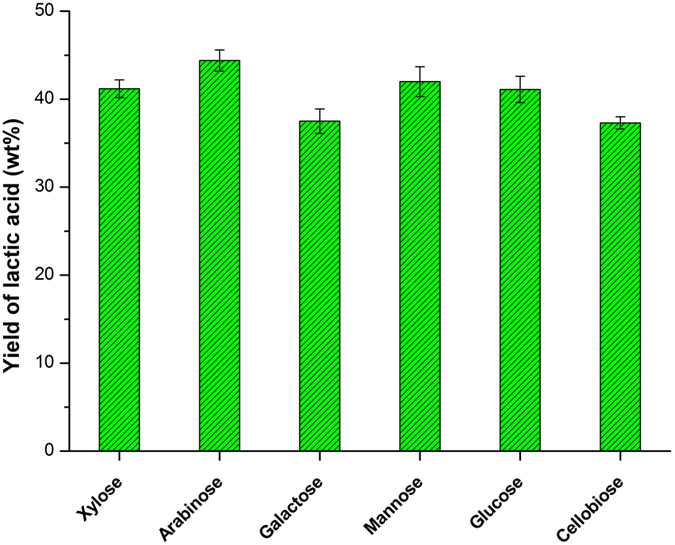
The yield of lactic acid derived from different sugars. Reaction condition: 0.63 g substrate, 0.5 g MgO, 220 °C, 1 h, initial pressure 2 MPa N_2_.

In most cases, the conversion of hemicellulose or xylan to lactic acid firstly passes through the depolymerization of hemicellulose or xylan to xylose monomer in the presence of acid catalyst. Then, the xylose is further converted to glycolaldehyde and glyceraldehyde via a retro-aldol condensation reaction. Consequently, lactic acid is produced^30^,^45^,^47^. In this reaction pathway, the theoretical yield of lactic acid based on xylose can be calculated as 60 wt%. In our work, the highest yield of lactic acid reached up to 79.6 wt% based on the mass of hemicellulose contained in corn stover, which was much higher than the theoretical yield. In order to investigate the origin of the high yield of lactic acid, Liquid B was analyzed by GPC. The results showed that there were still some oligomers (M_w_: 654 g mol^−1^) in Liquid B. Control experiments were therefore conducted using microcrystalline cellulose and cellobiose as the starting material, respectively. The yield of lactic acid derived from cellobiose was lower than 40 wt% (shown as Fig. 7), while lactic acid was hardly obtained from microcrystalline cellulose. Considering the fact that only 8 wt% of cellulose was converted, we believed that the majority of lactic acid was derived from hemicellulose. In addition, when other sugars contained in corn stover, including xylose, arabinose, glucose, glacatose and mannose, were employed as substrates under the same conditions of the second step (0.5 g MgO, 220 °C and 1 h), the yield of lactic acid was less than 45 wt% (Fig. 7). These results indicated that cellobiose and other sugars contained in corn stover were not mainly responsible for the high yield of lactic acid. Besides hemicellulosic/cellulosic oligomers, glucose and xylose, Liquid B also contained some lactic acid, FA and AA (Table 2) generated from the first step. It was therefore inferred that lactic acid, FA and AA might influence on the pathway of xylose conversion to lactic acid. Control experiments were also conducted, under the same conditions of the second step (0.5 g MgO, 220 °C and 1 h), using xylose as substrate with the addition of equivalent quantity of FA, AA and lactic acid in reaction system, respectively. When the additive was FA or AA, it generated <40 wt% of lactic acid. Unexpectedly, the addition of lactic acid significantly promoted the yield of lactic acid (>60 wt%), which was much higher than that without the addition of lactic acid (41 wt%). However, in the absence of MgO, the main product was ~30 wt% of furfural using xylose as substrat with the addition of FA and AA, respectively; and when the additive was lactic acid, no newly generated lactic acid was obtained. It suggested that the added-lactic acid and MgO may exhibit synergistic effect on the unusually high yield of lactic acid. Of course, it is necessary to deeply investigate in future work. We therefore inferred that the formation of lactic acid in the second step might pass through some other pathways different from the typical reaction mechanism reported^30^, for example, the formation of lactic acid directly from oligomers (similar to the formation of glycol directly from cellulose oligomers reported by Tao Zhang *et al*.^48^). In this case, the first step provided special feedstock for the second step to produce lactic acid with unusually high yield (79.6 wt%) catalyzed by MgO.

## Conclusions

We have achieved the fractionation of the three components in corn stover into aqueous hemicellulose derivatives, solid cellulose and solid lignin. The obtained hemicellulose derivatives, which was difficult to store and transport, was further converted to value-added chemical (lactic acid) with high yield (79.6 wt%) and selectivity (90%), through a well-designed chemo-catalytic biorefining process. Firstly, ~90 wt% of the hemicellulose and lignin in corn stover were simultaneously dissolved in ethanol/H_2_O (1/1, v/v) co-solvent with 0.050 M oxalic acid at 140 °C for 1 h; while the cellulose was not obviously degraded and could be separated by simple filtration. Wherein, H_2_O dissolved hemicellulose, G unit and those with β-O-4 linkage of lignin; while ethanol extracted G and S units as well as the skeleton with β-5 and β-β linkages of lignin. Oxalic acid effectively catalyzed the hydrolysis of hemicellulose and broke the intermolecular linkages between hemicellulose and lignin, and therefore further promoted the release of lignin. Then, the released lignin was precipitated after removal of ethanol. The obtained solid cellulose and lignin may be suitable raw materials for bio-chemicals and bio-oil production. Finally, the dissolved hemicellulose derivatives, co-existed as a mixture including xylose, cellulosic/hemicellulosic oligomers and lactic acid, was used as feedstock to enhance the yield of lactic acid with MgO at 220 °C. The first step (fractionation) provided special feedstock for the second step to produce lactic acid with unusually high yield catalyzed by MgO. MgO exhibited good recyclability. This comprehensive process may be very promising to effectively utilize the raw biomass to its fullest and produces target products with high selectivity.

## Method

### Materials

Corn stover was obtained from Fushun in Liaoning province of China. It was smashed by miniature plant sample mill (1400 r min^−1^, 180 W, 2 g min^−1^). The size of corn stover powder ranged from 60–100 mesh. It was dried in an oven at 100 °C overnight before use. The chemicals used in this work were obtained from commercial sources and used without further purification except MgO, which was calcined at 700 °C for 4 h before use.

### Simultaneous dissolution of hemicellulose and lignin in corn stover

The treatment of corn stover to simultaneously dissolve hemicellulose and lignin was performed in a 200 mL stainless steel autoclave equipped with a magnetic stirring device and a temperature controller. 3.0 g corn stover powder and designed amount of oxalic acid were added in the reactor with 100 mL ethanol/H_2_O (typically 1/1, v/v). Then nitrogen gas was bubbled into the autoclave for three minutes at a rate of 300–400 mL min^−1^, so that the interior air was completely replaced by N_2_. The initial pressure was kept at 2.0 MPa. The reactor was heated (4 °C min^−1^) from room temperature to the target temperature and kept for designed reaction time (typically 1 h). When the reaction was finished, the reactor was pulled out from the heating device and cooled down naturally to room temperature. After depressurizing the reactor, the mixture was fully poured out. The reactor was washed for three times using ethanol/H_2_O (1/1, v/v), and the solution was merged to the mixture. Then the solid residue was separated from liquid fraction by vacuum filtration, washed with ethanol/H_2_O (1/1, v/v) for three times and dried at 100 °C overnight. The washing liquor and liquid fraction were mixed together (defined as Liquid A) and stored for subsequent processing.

### Recovery of lignin

Rotary evaporator was used to evaporate ethanol from Liquid A. Distillation conditions: 34–37 °C, vacuum pressure 0.09 MPa, 150 rpm, 1 h. Then the dissolved lignin oligomers were precipitated and filtered. After drying naturally, a reddish brown powder was obtained. The remained aqueous solution was defined as Liquid B.

### Enhancement of lactic acid production

Liquid B was further subjected to enhance lactic acid production by the catalysis of MgO. Designed amount of Liquid B and MgO were placed in a steel autoclave, which was then sealed and pressurized with 2.0 MPa N_2_. The reactor was heated (4 °C min^−1^) from room temperature to the target temperature. After the reaction was finished, the reactor was pulled out from the heating device, and cooled down naturally to room temperature. The mixture in the reactor was poured out and filtered to recover the catalyst by vacuum filtration. The filter residue was washed with 15 mL water for 3 times. The filtrate and washing liquor were merged together and further characterized by HPLC. We performed the same experiment at least in triplicate for each run, and the standard error was <5%.

### Characterization of the solid residue

Classical chemical titration methods were employed to analyze the three main components (cellulose, hemicellulose and lignin) in corn stover and in the reaction residue. The experimental details could be found in refs 36 and 49. The FTIR spectra of the solid samples were recorded on a Nicolet 6700 Fourier transform infrared spectrometer in the range of 4000–400 cm^−1^ with a resolution of 4 cm^−1^. 1 mg dried samples were blended with 100 mg KBr and pressed into thin pellets before measurement.

### Characterization of liquid products

Qualitative and quantitative analyses of the small molecular liquid products were performed on the Dionex Ultimate 3000 high performance liquid chromatograph (column HPX-87H 300 × 7.8 mm) with a RI detector. The mobile phase was 0.005 M sulfuric acid aqueous solution with a flow rate of 0.6 mL min^−1^. The detection temperature of column and RI detector were 50 °C and 35 °C, respectively. The total testing time was 55 min. External standard method was employed to quantify the products, using calibration curves from authentic samples. The yield of liquid products and the selectivity to lactic acid reported in this work are defined as follow:


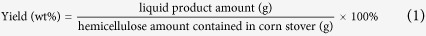



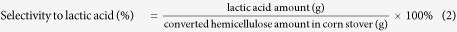


The weight-average molecular weights (M_w_) of liquid products and recovered lignin were analyzed using a gel permeation chromatograph (GPC, HLC-8320) with TSK gel Super HM-H (6.0 mm ×15 cm ×2) columns and a RI detector. Both the columns and RI detector were maintained at 40 °C during analysis. After removing solvents (H_2_O and ethanol) from the reaction liquid fraction, a viscous liquid was obtained as the sample. THF was used to dissolve the sample (for both recovered lignin and viscous liquid) and also used as eluent with a flow rate of 0.6 mL min^−1^ for GPC analysis. An injection volume of 10 μL was used. A calibration curve was obtained using monodisperse polystyrene standards. The 2D HSQC NMR spectra of liquid products from lignin were qualitatively determined on a Bruker Avance 400 MHz spectrometer. About 50 mg sample was fully dissolved in 0.5 mL deuterated dimethylsulfoxide (DMSO-d^6^). Heteronuclear single quantum coherence (HSQC) experiments were performed with 10 to 0 ppm and 220 to 5 ppm in ^1^H- and ^13^C-dimensions, respectively. For the ^1^H-dimension, 1024 data points were used for an acquisition time of 64 ms, while 256 data points were used for the ^13^C-dimension. The ^1^*J*_C–H_ used was 145 Hz. Besides, 1.5 s pulse delay and 16 scans were also adopted. The DMSO solvent peak (*δ*_C_ 39.5 ppm and *δ*_H_ 2.5 ppm) was used for the chemical shift calibration.

## Additional Information

**How to cite this article**: He, T. *et al*. Fractionation for further conversion: from raw corn stover to lactic acid. *Sci. Rep.* **6**, 38623; doi: 10.1038/srep38623 (2016).

**Publisher's note:** Springer Nature remains neutral with regard to jurisdictional claims in published maps and institutional affiliations.

## Supplementary Material

Supplementary Information

## Figures and Tables

**Table 1:** Effect of different reaction conditions on the conversion of corn stover[a].

Entry	Conditions		Conversion of corn stover[b]		
	CE[c]	COA[c]	Cellulose	Hemicellulose	Lignin
1	0.0	0.000	0.0	44.5	27.5
2	0.3	0.000	2.9	23.4	28.5
3	0.5	0.000	0.4	15.8	38.6
4	0.7	0.000	0.0	7.1	29.5
5	1.0	0.000	0.0	0.0	16.9
6	1.0	0.050	0.0	2.3	15.2
7	0.0	0.050	12.0	98.1	47.0
8	0.5	0.005	3.0	65.5	63.3
9	0.5	0.010	5.1	83.2	77.6
10	0.5	0.017	6.0	84.0	83.0
11	0.5	0.033	6.7	86.7	87.1
12	0.5	0.050	7.8	88.0	89.2
13	0.5	0.067	14.1	91.5	89.4

^[a]^The corn stover contains 45 wt% of cellulose, 21 wt% of hemicellulose,17 wt% of lignin, 9 wt% of extractives, 3 wt% of ash, and 5 wt% of unknown composition. Reaction conditions: 3.0 g corn stover, 100 mL solvent, 140 °C, 1 h, initial pressure 2 MPa N_2_.

^[b]^Conversion percentage of the component based on its mass content in raw corn stover (wt%).

^[c]^Abbreviations used: CE = the concentration of ethanol in water (v/v); COA = the concentration of oxalic acid in 100 mL solvent (M).

**Table 2:** The yield of main liquid products and the selectivity to lactic acid in Liquid B and further reaction at different temperature.

Temperature (°C)	Yield wt%					SL[b]
	Xylose	Lactic acid	FA	AA	Furfural	
**Liquid B**	43.3	39.5	6.7	7.5	0.6	45
160[a]	50.9	44.9	11.6	7.9	2.7	51
180[a]	34.9	39.0	12.6	9.5	8.4	44
220[a]	1.2	14.5	8.8	11.2	21.0	16

^[a]^Reaction conditionns: 1 h without the addition of catalysts, initial pressure 2 MPa N_2_.

^[b]^SL = the selectivity to lactic acid (%).
