# Supplementary material for: Fractionation for further conversion: from raw corn stover to lactic acid
Source: Sci Rep. 2016 Dec 5;6:38623. doi: 10.1038/srep38623 (PMC5137029; doi:10.1038/srep38623)
Supplement: Supplementary Information [file srep38623-s1.pdf]

-Supplementary Information-

Fractionation for further conversion: from raw corn stover to lactic acid

Ting He, Zhicheng Jiang, Ping Wu, Jian Yi, Jianmei Li* and Changwei Hu*

Key Laboratory of Green Chemistry and Technology, Ministry of Education, College of Chemistry, Sichuan University, Chengdu 610064, P. R. China.

Received date; Fax: +86 28 85411105; Tel: +86 28 85411105.

E-mail: changweihu@scu.edu.cn, lijianmei@scu.edu.cn

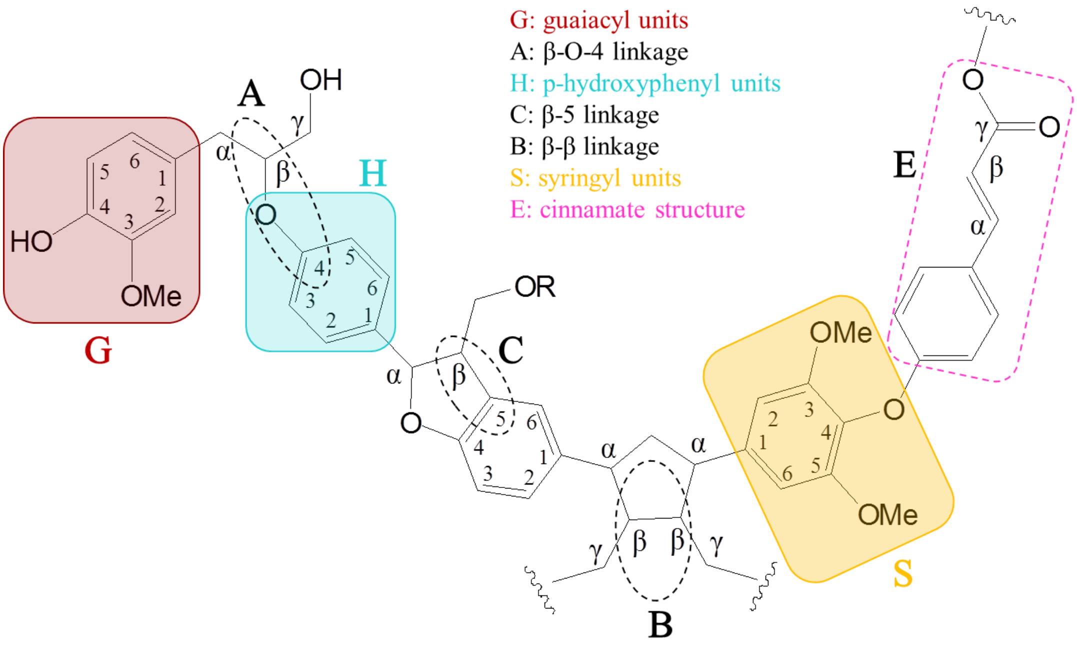

Figure S1. The hypothetical structure of partial lignin.

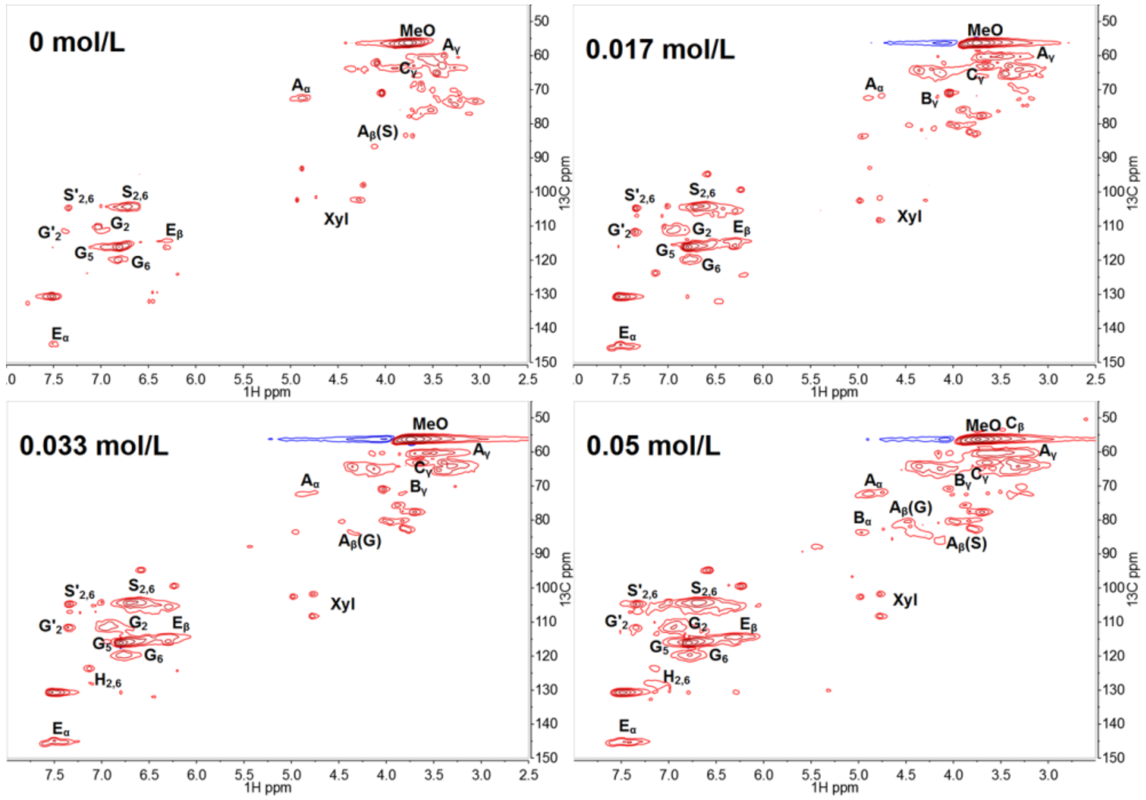

Figure S2. 2D HSQC NMR spectra of the liquid fractions obtained from the treatments with different concentration of oxalic acid in co-solvent. Reaction condition: 3.0 g corn stover, 100 mL ethanol/H_2O (1/1 v/v), 140 ℃, 1 h, initial pressure 2 MPa N_2. A: β-O-4 linkage; B: β-β linkage; C: β-5 linkage; E: cinnamate structure; G: guaiacyl units; S: syringly units; H: p-hydroxyphenyl unit; MeO: methoyls; Xyl: xylose derived from hemicellulose.

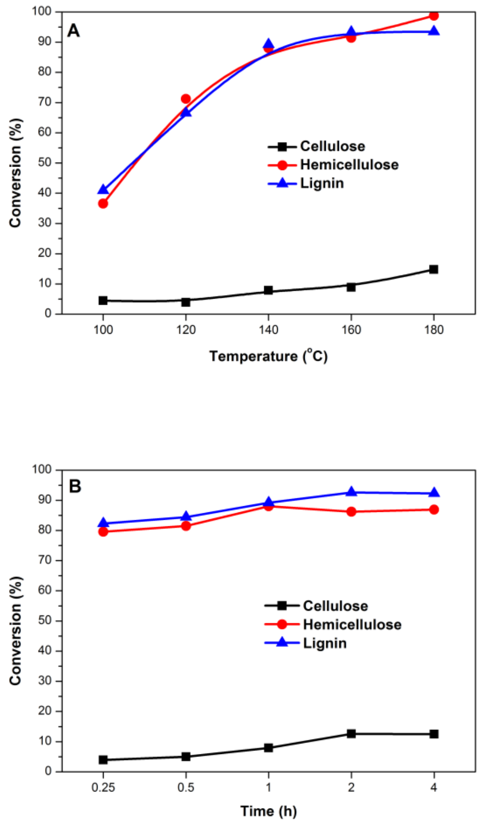

Figure S3. The influence of different reaction temperature (A) and time (B) on the conversion of three components in corn stover. Reaction condition: 3.0 g corn stover, 100 mL ethanol/H_2O (1/1 v/v), initial pressure 2 MPa N_2.

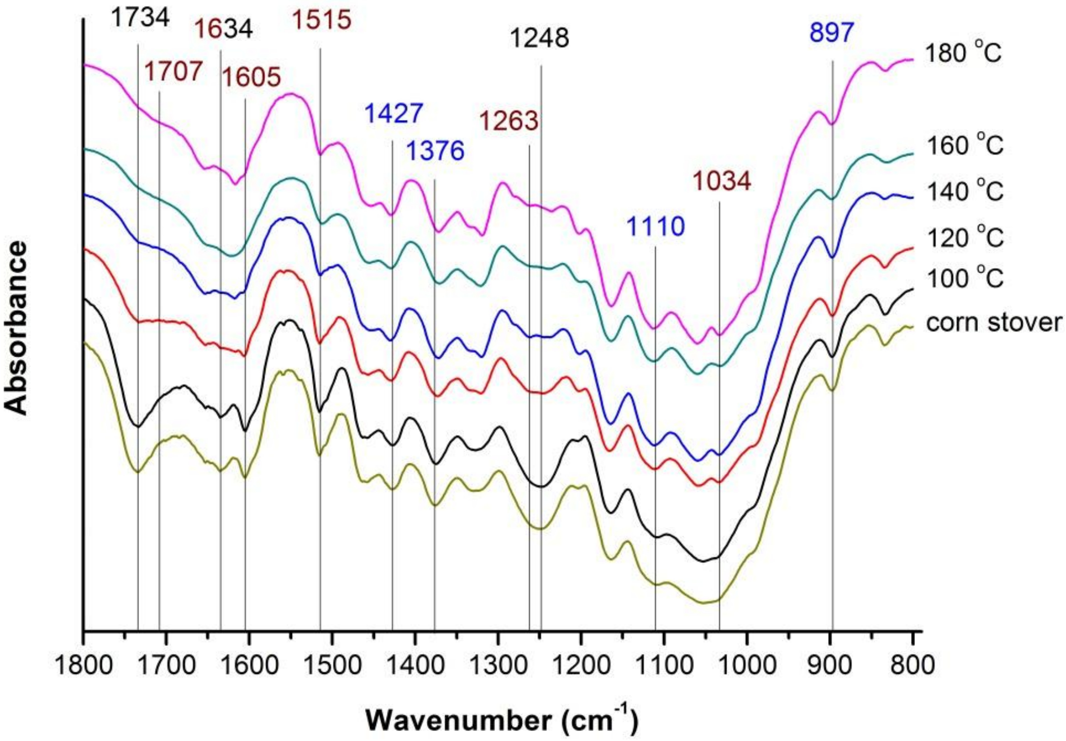

Figure S4. FTIR spectra of solid samples obtained at different treatment temperatures.

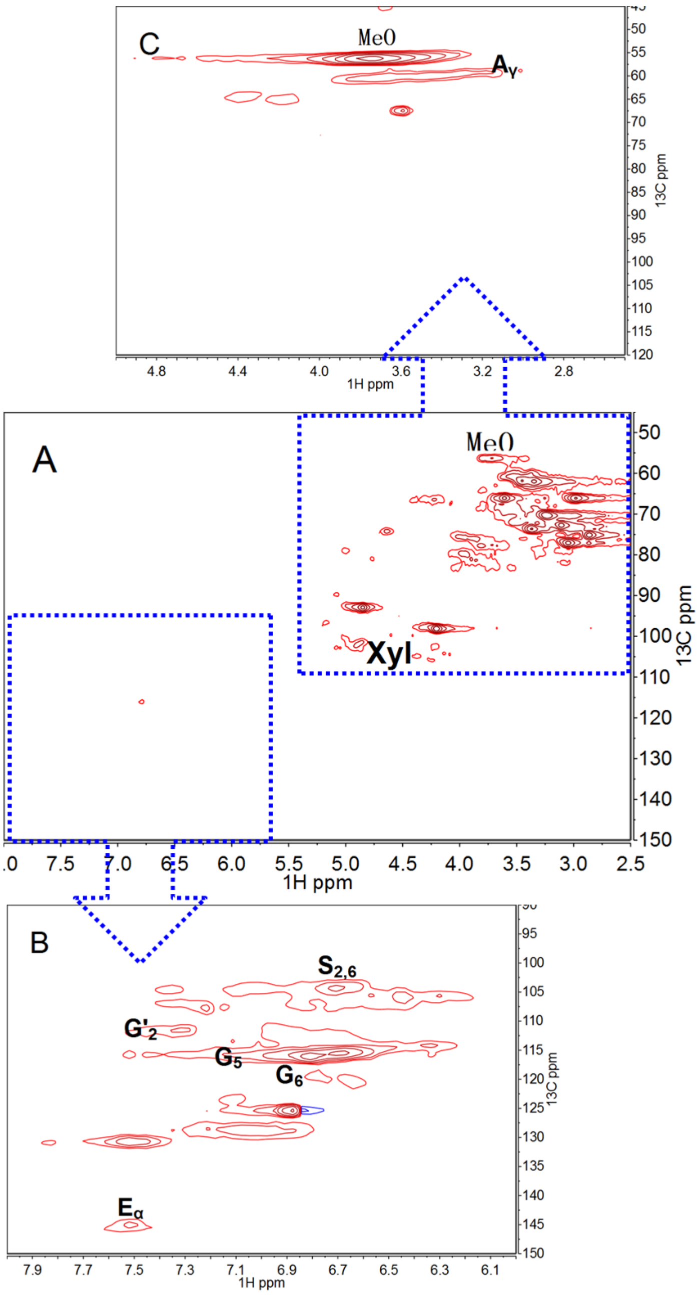

Figure S5. 2D HSQC NMR of the samples obtained in water with 0.05 M oxalic acid.

The liquid fraction obtained in water with 0.05 M oxalic acid was treated by the same way that was mentioned in **Method section** in Manuscript before detection and obtained (A) spectra. Due to the thimbleful content in the sample, the signal of lignin oligomers was too weak to be observed. In order to investigate the solvent properties on lignin dissolution, we extracted the lignin oligomers in the liquid fraction by THF-NaCl system. After removal of THF, the obtained sample was also conducted to 2D HSQC characterization, and the result was presented in aromatic C-H correlation region (B) and aliphatic C-H correlation region (C).

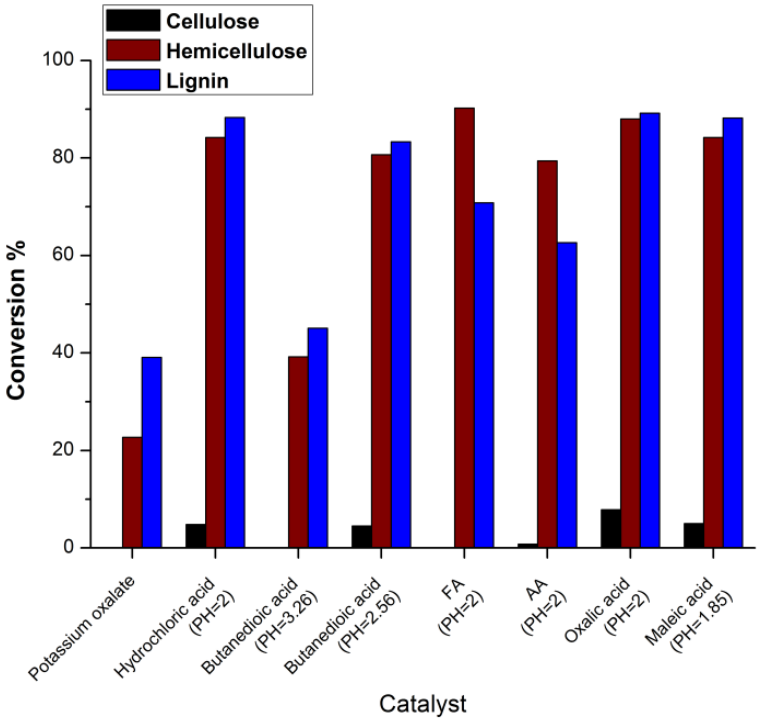

Figure S6. The conversion of three components in corn stover with different catalysts. Reaction conditions: 3.0 g corn stover, 100 ml ethanol/H_2O (1/1, v/v), 140 ℃, 1 h, initial pressure 2 MPa N_2.

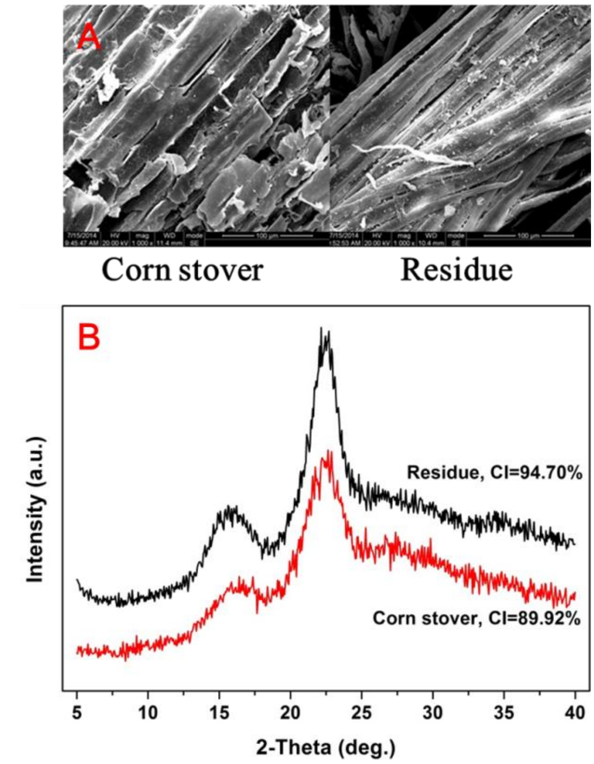

Figure S7. SEM and XRD graphs of raw corn stover and reaction residue.

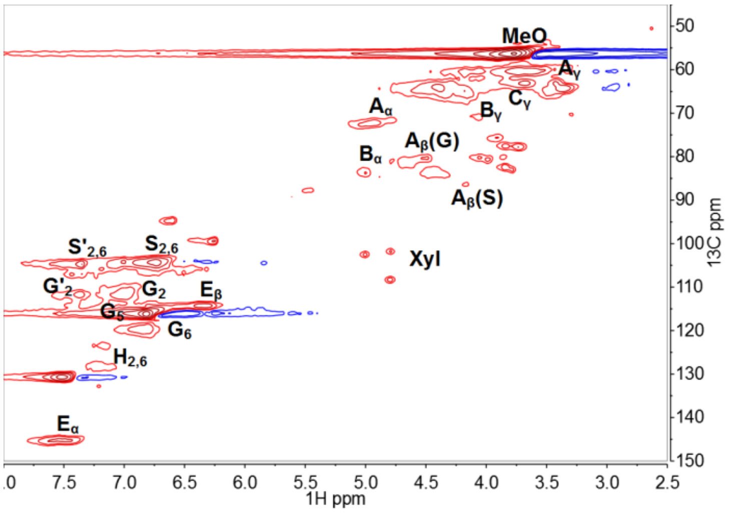

Figure S8. 2D HSQC of the recovered lignin.

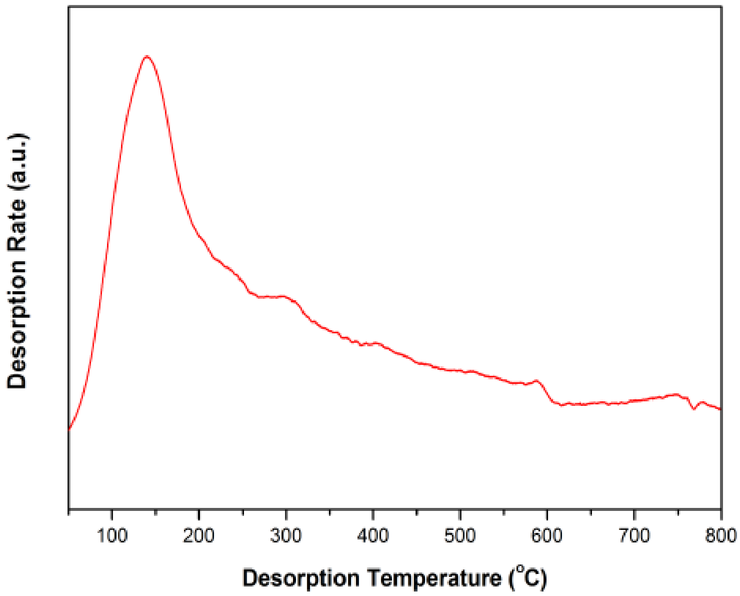

Figure S9. The CO_2 desorption analysis of MgO.

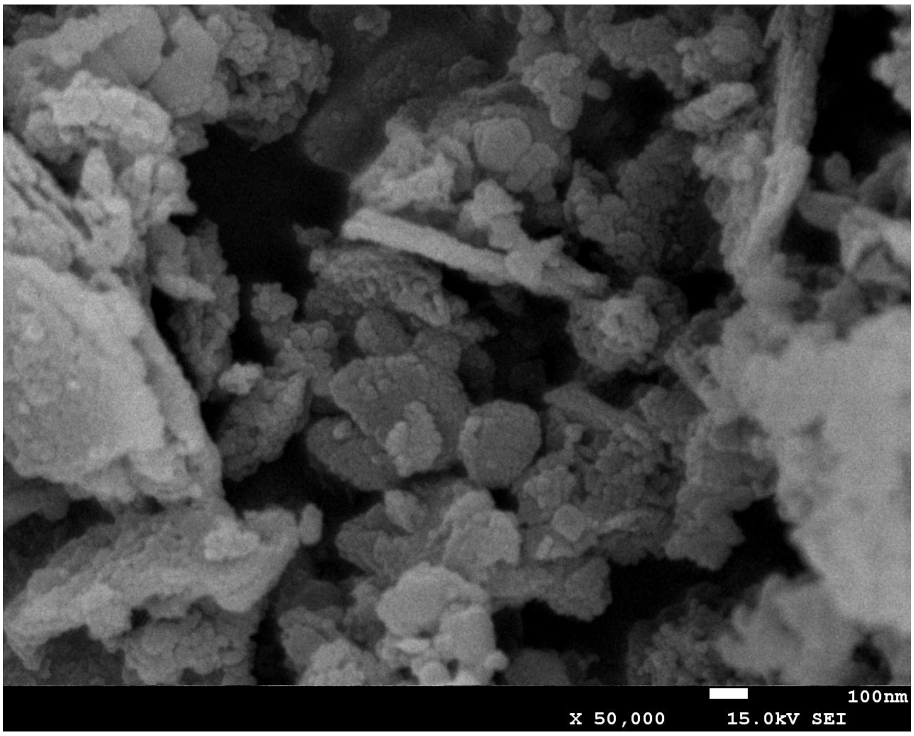

Figure S10. The SEM of fresh MgO.

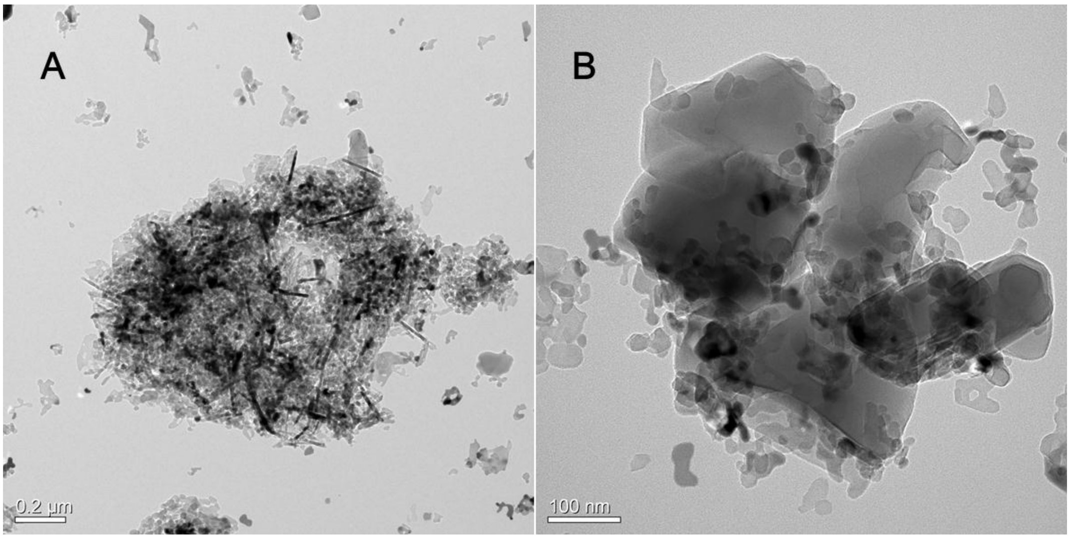

Figure S11. The TEM of fresh MgO.

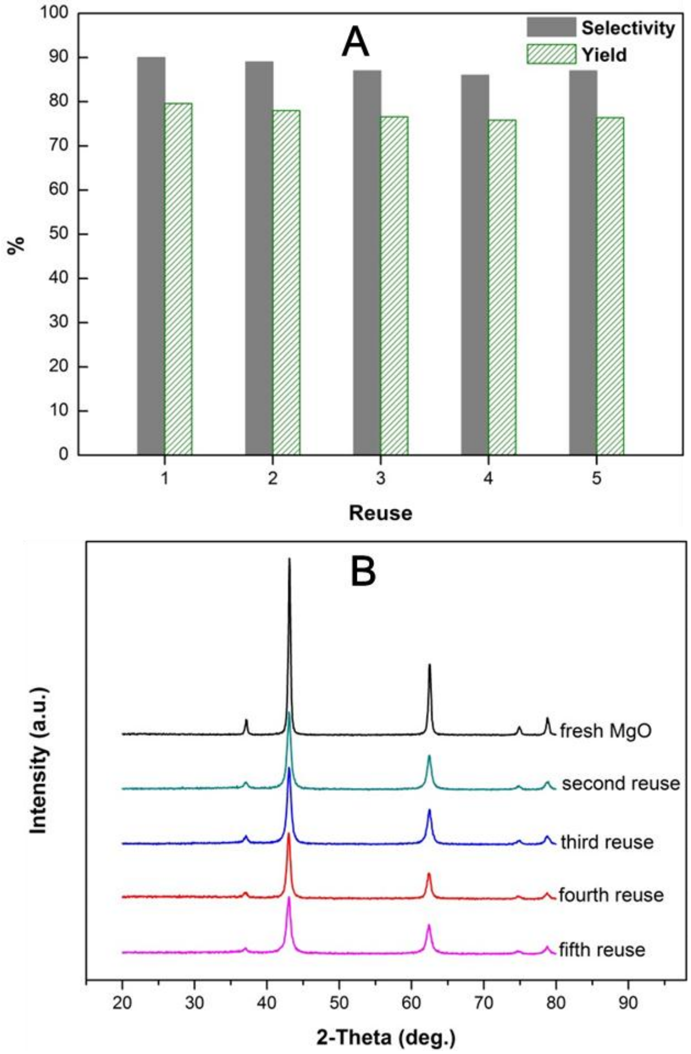

Figure S12. (A) Reuse of MgO for the production of lactic acid. (B) XRD graphs of fresh and reused MgO.

Table S1 Assignment of main lignin ^{13}C-^{1}H correlation signals in HSQC spectra of liquid fraction according to the literature

Labels	δ_C/δ_H (ppm)	Assignment
MeO	56.0/3.71	C-H in methoyls
A_α	72.4/4.85	C_α-H_α in β-O-4 structures (A)
A_β(G)	84.9/4.32	C_β-H_β in β-O-4 structures linked to a G unit (A)
A_β(S)	86.2/4.11	C_β-H_β in β-O-4 structures linked to a S unit (A)
A_γ	59.8-60.2/3.23-3.71	C_γ-H_γ in β-O-4 structures (A)
B_α	84.6/4.66	C_α-H_α in β-β structures (B)
B_γ	71.3/4.18, 3.82	C_γ-H_γ in β-β structures (B)
C_β	52.8/3.47	C_α-H_α in β-5 structures (C)
C_γ	62.7/3.73	C_γ-H_γ in β-5 structures (C)
E_α	144.5/7.50	C_α-H_α in cinnamate structures (E)
E_β	113.7/6.30	C_α-H_α in cinnamate structures (E)
G_2	111.2/7.00	C_2-H_2 in guaiacyl units (G)
G'_2	111.5/7.35	C_2-H_2 in oxidized (C_α=O) guaiacyl units (G)
G_5	114.9-115.9/6.75	C_5-H_5 in guaiacyl units (G)
G_6	119.5/6.83	C_6-H_6 in guaiacyl units (G)
$S_{2,6}$	104.4/6.72	$C_{2,6}$-$H_{2,6}$ in syringyl units (S)
$S'_{2,6}$	106.4/7.30	$C_{2,6}$-$H_{2,6}$ in (C_α=O) syringyl units (S)
$H_{2,6}$	128.2/7.19	$C_{2,6}$-$H_{2,6}$ in *p*-hydroxyphenyl units (H)
Xyl	102-110/4.0-5.5	C-H in xylose derived from hemicellulose
